# Development of a novel selective medium for culture of Gram-negative bacteria

**DOI:** 10.1186/s13104-021-05628-2

**Published:** 2021-05-29

**Authors:** Shooq Yousef Al-blooshi, Mustafa Amir Abdul Latif, Nour K. Sabaneh, Michael Mgaogao, Ashfaque Hossain

**Affiliations:** 1 Department of Medical Microbiology and Immunology, RAK Medical and Health Sciences University, Ras Al Khaimah, United Arab Emirates; 2 Central Research Laboratory, RAK Medical and Health Sciences University, Ras Al Khaimah, United Arab Emirates

**Keywords:** Mueller Hinton agar, Gram-positive bacteria, Gram-negative bacteria, Bacterial culture media, Selective media, Clove

## Abstract

**Objective:**

Although many bacterial culture media are available commercially, there is a continuous effort to develop better selective media for bacteria, which cannot be grown on existing media. While exploring antibacterial properties of clove, we observed that it has the potential to selectively inhibit growth of certain types of bacteria. This led us to do the experiments, which resulted in developing a new media which selectively allowed the growth of only Gram-negative bacteria, while inhibiting the Gram-positive bacteria.

**Results:**

Mueller Hinton Agar (MHA) was used as the base media and was modified to develop MHA-C15 (MHA containing 15% volume/volume water extract of clove). Gram-negative bacterial pathogens *Escherichia coli*, *Klebsiella pneumoniae*, *Salmonella typhimurium* and *Pseudomonas aeruginosa* grew on MHA-C15. However, none of the major Gram-positive bacterial pathogens such as *Staphylococcus aureus*, *Streptococcus pneumoniae*, *Streptococcus pyogenes*, *Streptococcus mutans, Bacillus *spp. and *Enterococcus *spp. grew on it. Taken together, these findings show that MHA-C15 is a newly developed selective media for culture of Gram-negative bacteria.

**Supplementary Information:**

The online version contains supplementary material available at 10.1186/s13104-021-05628-2.

## Introduction

Bacterial culture (growth) media contains nutrients necessary for their growth. All microorganisms cannot grow in a single culture medium as their growth requirements vary; while for many microorganisms, the growth requirements are unknown [1, 2]. Selective media allows the growth of one class of bacteria while inhibiting the others. For example, MacConkey agar is a selective media that inhibits the growth of many Gram-positive bacteria and favors the growth of Gram-negative bacteria, particularly the *Enterobacteriaceae* group of bacteria [3]. The use of selective media is essential in isolation of pathogens from infection sites so that accurate pathogen identification and diagnosis can be made and treatment can be initiated. In addition, it is an essential first step in microbiological investigation of environmental samples. Although several selective media are commercially available, there is a constant effort to develop newer media for more efficient isolation and identification of bacterial species [4]. Human body harbors an astonishingly high number of bacterial species [5]. Studies have shown that we have approximately the same or more number of bacteria in and on our body as compared to our own body cells [6]. Most of these bacterial species cannot be cultured in the commercially available culture media [7]. It is also predicted that a large number of bacteria species in the environment also cannot be cultured due to the lack of appropriate media which are needed for their growth [6]. This is the main driving force behind the constant effort for the formulation and development of newer media for culture of bacteria [8–10]. Such new media will allow scientists to grow and study currently un-culturable bacterial species.

Plants are rich in a wide variety of chemical compounds such as tannins, terpenoids, alkaloids, and flavonoids which have been found to possess antimicrobial properties against wide variety of bacterial pathogens. Since prehistoric times, traditional healers have used plants to prevent or cure infectious conditions [11]. Cloves are aromatic flower buds of the tree, *Syzygium aromaticum*. They have been widely known for their antibacterial, antiviral and antifungal properties and also for their use in herbal medicine for centuries. Cloves are used as a spice in cooking different types of food items and also used in food preservations for its antimicrobial effect with no known side effects. Clove essential oil (CEO) is traditionally used in the treatment of burns and wounds, and as a pain reliever in dental care as well as treating tooth infections and toothache [12]. The extracts of clove exhibit antimicrobial effect on multidrug resistant microorganisms as well as methicillin-resistant *Staphylococcus aureus, Bacillus subtilis, Salmonella typhi* and *Serratia marcescens*. Various phytochemicals such as sesquiterpenes, monoterpenes, hydrocarbon, and phenolic compounds are present in cloves. In clove oil, eugenyl acetate, eugenol, and β-caryophyllene are the most important phytochemicals exhibiting antibacterial activity [13].

Although different chemical compounds are being used to develop different types of selective media, surveys of existing literature showed that plant extracts have not been investigated extensively as a component in bacterial culture media. In this project, we used water extract of the clove to develop a selective media for Gram-negative bacterial species, which does not allow the growth of Gram-positive bacteria. It is anticipated that the finding of this research project will stimulate exploration of different plant materials for their suitability in developing selective and differential media for the growth of different types of bacteria present in nature for which there exists no appropriate culture media at present.

## Main text

### Materials and methods

#### Preparation of clove extract

Dried and powdered clove (Al Faris Spices, Salmabad, Bahrain) was used to prepare a water extract. A 20% (weight/volume) clove powder suspension in hot distilled water was prepared and mixed using a magnetic stirrer hot plate for 30 min at 50 °C. Then, the extracted material was filtered using Whatman filter paper and stored at 4 °C until used. Mueller Hinton agar (MHA) containing different concentrations of clove extract (5–20%) was prepared by adding different volumes of the clove extract and autoclaved. We followed the manufacturer’s instruction in preparation of the MHA plates and the volume of water to be added to the media to be prepared was adjusted according to the volume of clove extract to be added for each concentrations of extract. We labelled the plates MHA-C5 (MHA containing 5% extract volume/volume); MHA-C10 (MHA containing 10% extract volume/volume) and so on for other concentrations.

#### Inoculation of different bacteria

Gram-positive bacterial species tested were *Staphylococcus aureus, Staphylococcus epidermis, Streptococcus pneumoniae, Streptococcus pyogenes, Enterococcus faecalis*, *Bacillus subtilis* spp. and *Streptococcus mutants*. The Gram-negative bacterial species included in this study were *Escherichia coli, Klebsiella pneumoniae, Pseudomonas aeruginosa* and *Salmonella typhimurium*. Bacterial strains were grown overnight in Mueller Hinton broth. A loop-full of bacteria were taken from such cultures and streaked onto the MHA plates and on MHA containing different concentrations of extracts i.e., MHA-C5, MHA-C10, MHA-C15 and MHA-C20 and incubated at 37 °C for 24 h. The plates were then observed for growth.

### Results and discussion

In a study to determine antibacterial effect of clove extract on different bacteria, we serendipitously decided to incorporate clove extract into agar media. We used MHA in this study as this agar media is recommended for antibacterial susceptibility assays. Moreover, we were using this media in our initial experiments to determine the antimicrobial activity of clove extract. We observed that modified MHA (MHA containing clove extract) exhibited a differential property i.e., at certain concentration (20%) it inhibited the growth of Gram-positive bacteria *S. aureus*, but had no effect on the growth of Gram-negative bacteria, *E. coli*. We then tried a series of different MHA-clove plates with 5% increment in clove concentration (0%, 5%, 10%, 15% and 20%) in parallel to determine the minimum concentration of clove extract exhibiting this differential effect, i.e., inhibiting the growth of *S. aureus* but allowing the growth of *E. coli*. We observed from this experiment that MHA-C15 (MHA containing 15% clove extract) was the agar plate containing minimum concentration of clove extract exhibiting this differential effect i.e., allowing growth of *E. coli* but suppressing the growth of *S. aureus*. MHA-Clove-10 and MHA-Clove-5 supported the growth of both *S. aureus* and *E. coli*.

**Fig. 1:**
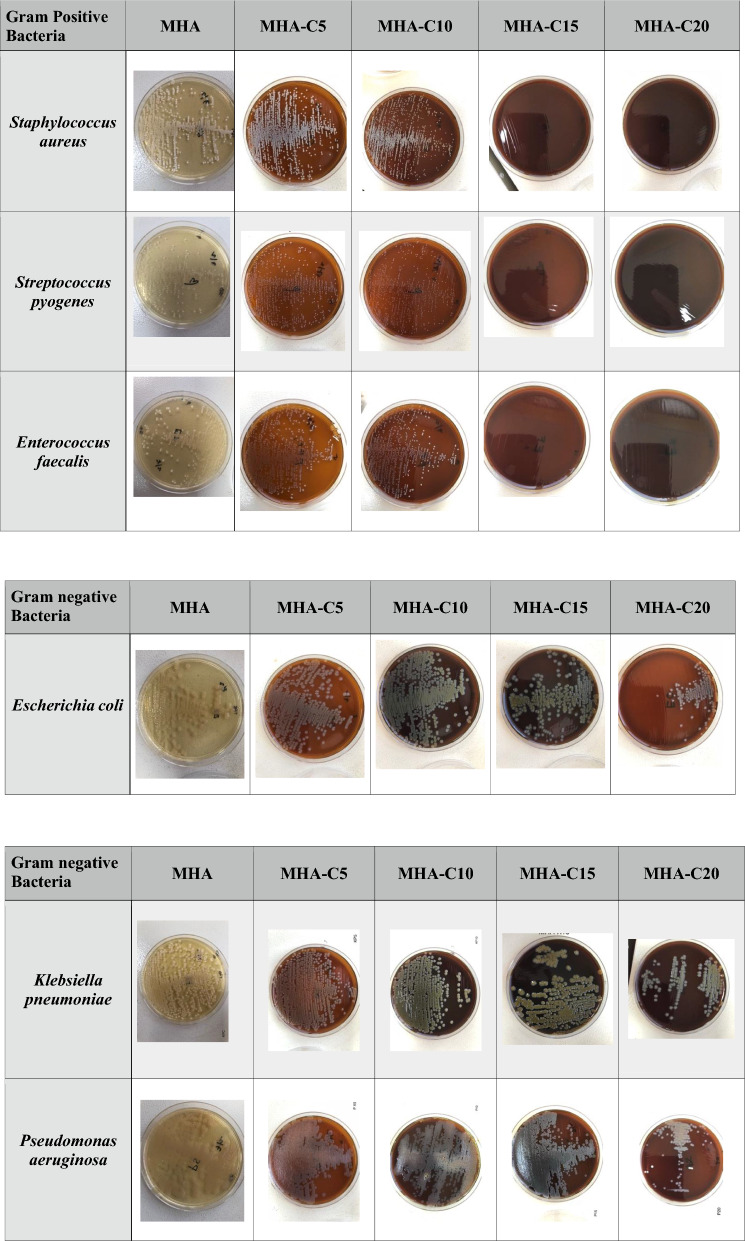
Growth of Gram-positive and Gram-negative bacteria on MHA and MHH containing different concentrations of extract (5%; MHA-C5 through 20%; MHA-C20). Gram-positive bacteria *Staphylococcus aureus*, *Enterococcus faecalis*, and *Streptococcus pyogenes* grew on MHA-C5 and MHA-C10 but not on MHA-C15 and MHA-C20. Gram-negative bacteria *Escherichia, Pseudomonas aeruginosa* and *Klebsiella pneumoniae* grew on MHA containing all the concentrations of extract tested

**Fig. 2:**
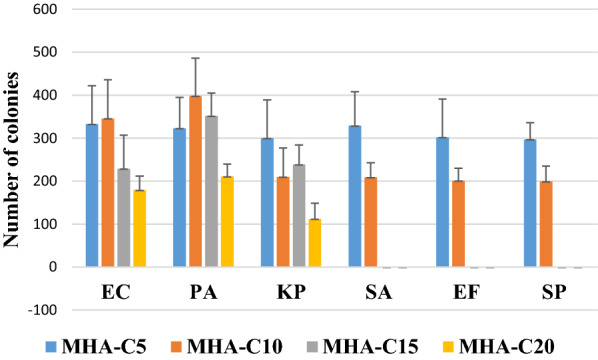
Growth of Gram-positive and Gram-negative bacteria on MHA containing different concentrations of clove extract. The data represents mean and standard error of mean of three independent experiments. MHA-C5 contains 5% of extract and so on (detailed in the materials and methods section). MHA-C15 had the lowest concentration the extract, supporting the growth of the Gram-negative bacteria while suppressing the growth of Gram-positive bacteria. *EC* *Escherichia coli*, *PA* *Pseudomonas aeruginosa*, *KP* *Klebsiella pneumonia*, *SA* *Staphylococcus aureus*, *EF* *Enterococcus faecalis*, *SP* *Streptococcus pyogenes*

After standardizing the concentration of incorporated clove extract needed to differentiate between major Gram-positive and Gram-negative bacterial species, we extended our study to include other Gram-positive and Gram-negative pathogens. To our great satisfaction, we observed that MHA-C15 which differentiated between growth of *S. aureus* and *E. coli*, also differentiated other Gram-positive and Gram-negative bacterial species (Figs. 1, 2). We tried MHA-C25 and observed that it inhibited the growth of both Gram positive and Gram-negative bacterial species. It is possible that at 25%, clove extract reaches toxic levels for both Gram-positive and Gram-negative bacterial species. Even on MHA-C15, the growth of Gram-negative bacteria was relatively less in comparison to MHA-C10 (Fig. 2), suggesting that clove extract has a concentration dependent inhibitory effect on bacterial growth.

The count of bacteria grown on different MHA-C plates are shown in Fig. 2. Counting of bacteria was carried out for quantitative evaluation of bacterial growth as a function of the concentration of the clove extract. Bacterial numbers decreased as the concentration of clove extract increased in the MHA plates for both Gram-positive and Gram-negative bacteria. However, on MHA-C15, the count of Gram- positive bacteria dropped to zero, but Gram-negative organisms continued to grow. Comparison of relative growth of three major Gram-negative bacteria on MHA-C15 showed that *Pseudomonas aeruginosa* grew best, which was followed by *Escherichia coli* and *Klebsiella pneumoniae*. Opportunistic bacteria *Pseudomonas aeruginosa* is intrinsically resistant to different antimicrobial agents because of the presence of highly efficient efflux systems which permits its growth in presence of different inhibitors [14]. This capacity may have contributed to its efficient growth on MHA-C15. We carried out Gram staining and antibiotic sensitivity testing (by disk diffusion method) of bacteria grown MHA and MHA-C15 grown in parallel. No changes were noticed either in Gram staining or in antibiotic sensitivity of the organisms, indicating that MHA-C15 can be used to cultivate bacteria with no apparent changes in these vital properties of the bacteria tested (Additional file 1: Figures S1, S2, S3, S4).

Other Gram-positive and Gram-negative bacteria tested included *Bacillus* and *Streptococcus mutans* and *Salmonella typhimurium.* These bacteria also followed the same pattern i.e., growth on MHA-C15 for Gram-negatives and no growth for Gram-positives (data not shown). This finding demonstrates that the concentration of the clove present in these two media (MHA-C5 and MHA-C10) was not inhibitory and allowed the growth of all the bacterial species tested. However, no growth was observed on MHA-C15 and MHA-C20 for all the Gram-positive bacterial species, indicating that the clove present in this medium inhibited growth of these bacteria.

These data demonstrate that MHA-C15 and MHA-C20 are the media that do not allow growth of Gram-positive bacteria tested in this study but allows the growth of Gram-negative bacteria. However, the number of colonies on MHA-C20 decreased for Gram-negative bacteria compared to MHA-C15. So, MHA-C15 was media containing the lowest concentration of extract which supported the growth of Gram-negative bacteria but inhibited the growth of Gram-positive bacteria. Taken together, MHA-C15 may be considered as an ideal media for Gram-negative. However, investigation with other Gram-negative bacteria needs to be done to determine whether the observation with the selected Gram-negative bacteria also holds true for other bacterial species belonging to this group of bacteria.

How clove extract selectively inhibits Gram-positive bacteria is unknown. The antimicrobial activity of clove extract is reported to be associated with Eugenol (2 methoxy-4 allyl-phenol), the main component of clove oil, which is known to exhibit antibacterial and antifungal activity. Antimicrobial activity of clove also reported to be due to high tannin content (10–19%) [15–17]. The cell wall of Gram-positive bacteria has a thick layer of peptidoglycan, which is much thinner in Gram-negative bacteria. This difference in cell wall thickness is basis for differential susceptibility of many bacterial species to different type of antibiotics and natural compounds. So, it may be speculated that the primary target for the growth inhibitory compounds presents in clove is most likely bacterial cell wall [18]. The culture of microorganisms is a prerequisite for any study with them. Bacterial community in environment or in clinical settings is usually polymicrobial, consisting of both Gram-positive and Gram-negative species. It is often challenging to differentiate the different types of bacteria and grow them in pure culture. As this newly developed MHA-C15 selective medium differentiates between Gram-positive and Gram-negative bacterial species, it will serve as a useful adjunct to the currently available bacterial culture media.

In conclusion, after several trials with different concentrations of water extract of clove, we found that MHA-C15, supported the growth of different Gram-negative bacterial species and at the same time inhibited the growth of all the Gram-positive bacterial species tested. So, MHA-C15 can be described as a culture media for selective growth of Gram-negative bacteria. It is anticipated that this newly developed media would prove useful in the selective culture of other Gram-negative bacterial species in both clinical and environmental settings. Our future goal is to use graded concentration of clove extract with and without other plant materials to formulate bacterial growth medium which will allow differential growth of different species of Gram-negative bacteria.

### Limitations

Limited number of pathogenic Gram-positive and Gram-negative bacterial species were tested.

## Supplementary Information

**Additional file 1**: **Figure S1.** Gram staining of *Escherichia coli *(EC) grown on (a) MHA, (b) MHA-C15; antibiotic sensitivity test of EC grown on (c) MHA and (d) MHA-C15. **Figure S2.** Gram staining of *Klebsiella pneumoniae *(KP) grown on (a) MHA, (b) MHA-C15; antibiotic sensitivity test of KP grown on (c) MHA and (d) MHA-C15. **Figure S3.** Gram staining of *Pseudomonas aeruginosa *(PA) grown on (a) MHA, (b) MHA-C15; antibiotic sensitivity test of PA grown on (c) MHA and (d) MHA-C15. **Figure S4.** Gram staining of *Staphylococcus aureus *(SA) grown on (a) MHA, (b) MHA-C15; antibiotic sensitivity test of SA grown on (c) MHA and (d) MHA-C15.

## Data Availability

Additional files available. The datasets used and/or analysed during the current study available from the corresponding author (ashfaque@rakmhsu.ac.ae) on reasonable request.

## Abbreviations

EC: *Escherichia coli*
KP: *Klebsiella pneumoniae*
PA: *Pseudomonas aeruginosa*
SA: *Staphylococcus aureus*
MHA: Mueller Hinton agar
MHA-C15: MHA containing 15% clove extract
CEO: Clove essential oil
