# Supplementary material for: Development of a novel selective medium for culture of Gram-negative bacteria
Source: BMC Res Notes. 2021 May 29;14:211. doi: 10.1186/s13104-021-05628-2 (PMC8164760; doi:10.1186/s13104-021-05628-2)
Supplement: Supplementary file 1 — Additional file 1: Figure S1. Gram staining of Escherichia coli (EC) grown on (a) MHA, (b) MHA-C15; antibiotic sensitivity test of EC grown on (c) MHA and (d) MHA-C15. Figure S2. Gram staining of Klebsiella pneumoniae (KP) grown on (a) MHA, (b) MHA-C15; antibiotic sensitivity test of KP grown on (c) MHA and (d) MHA-C15. Figure S3. Gram staining of Pseudomonas aeruginosa (PA) grown on (a) MHA, (b) MHA-C15; antibiotic sensitivity test of PA grown on (c) MHA and (d) MHA-C15. Figure S4. Gram staining of Staphylococcus aureus (SA) grown on (a) MHA, (b) MHA-C15; antibiotic sensitivity test of SA grown on (c) MHA and (d) MHA-C15. [file 13104_2021_5628_MOESM1_ESM.pdf]

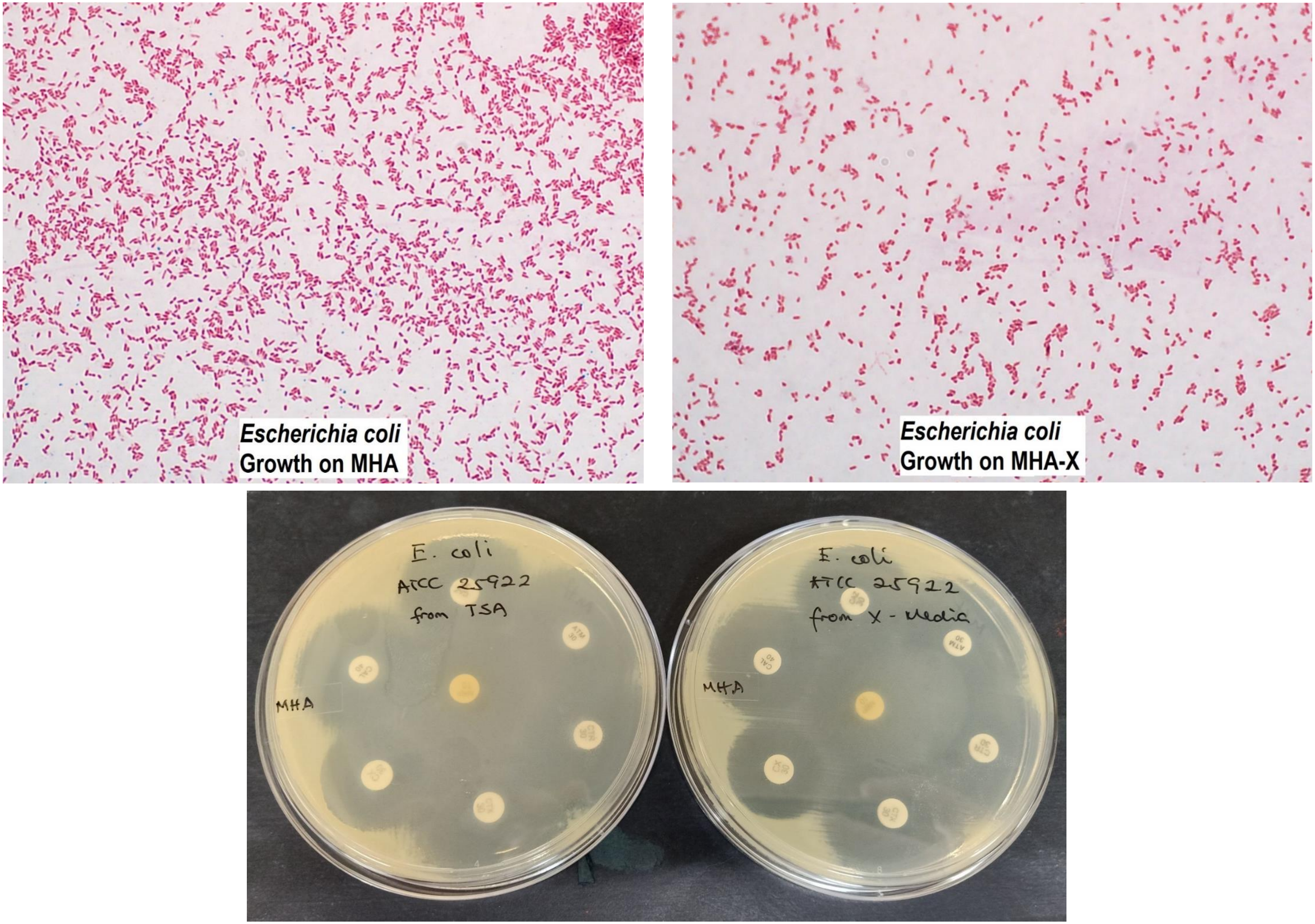

Figure 3. Gram staining of *Escherichia coli* (EC) grown on (a) MHA, (b) MHA-C15; antibiotic sensitivity test of EC grown on (c) MHA and (d) MHA-C15.

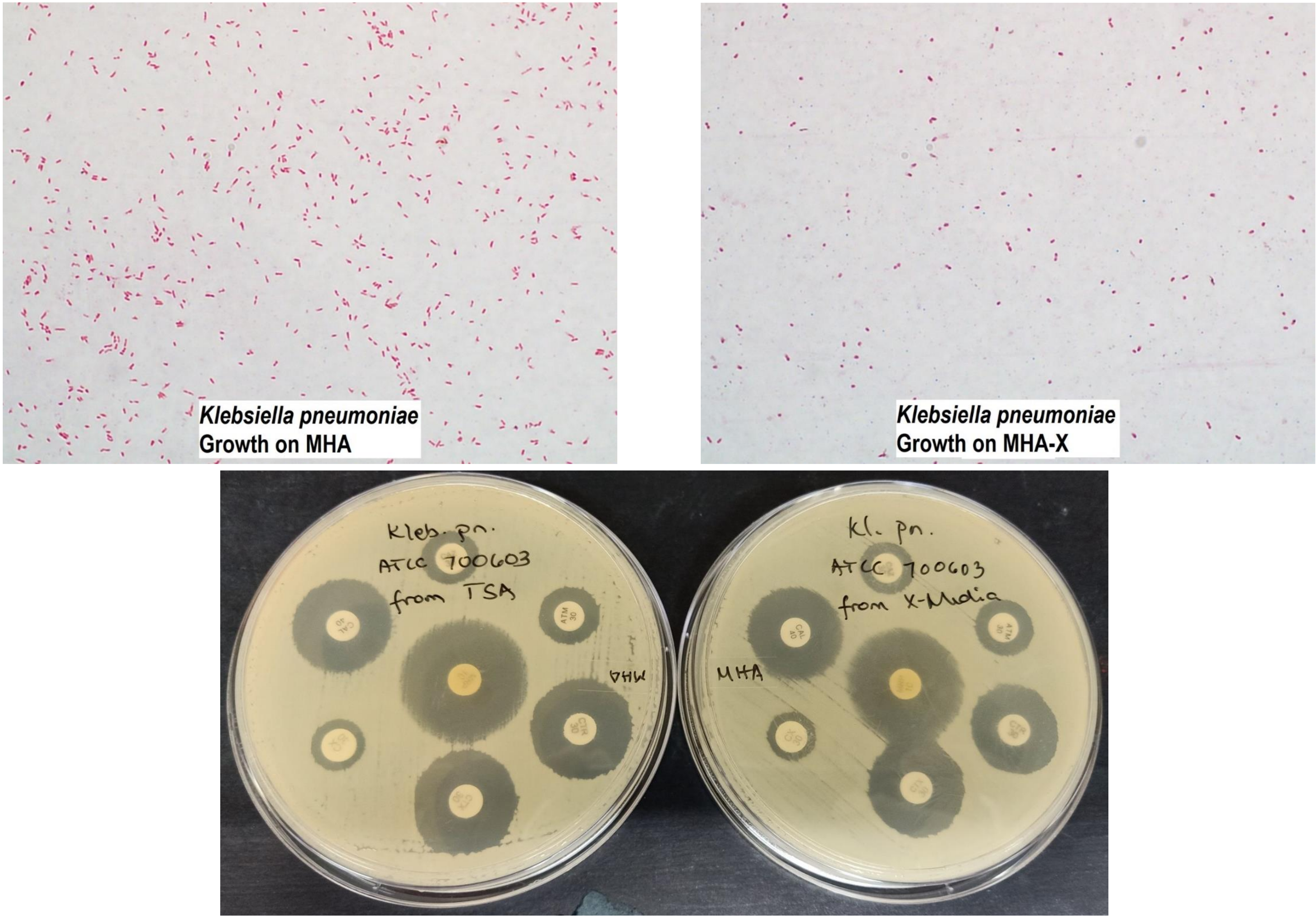

Figure 4. Gram staining of *Klebsiella pneumoniae* (KP) grown on (a) MHA, (b) MHA-C15; antibiotic sensitivity test of KP grown on (c) MHA and (d) MHA-C15.

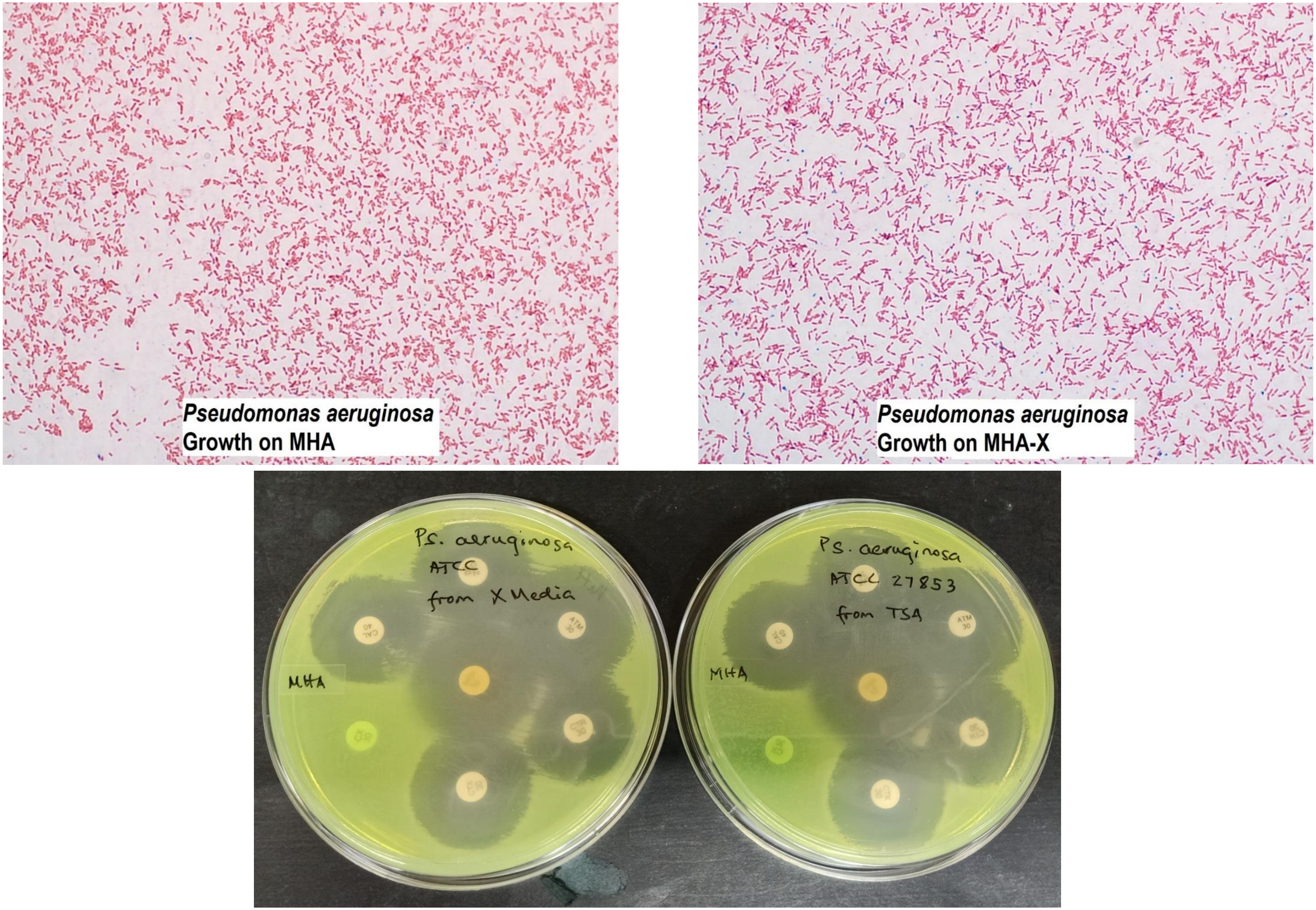

Figure 5. Gram staining of *Pseudomonas aeruginosa* (PA) grown on (a) MHA, (b) MHA-C15; antibiotic sensitivity test of PA grown on (c) MHA and (d) MHA-C15.

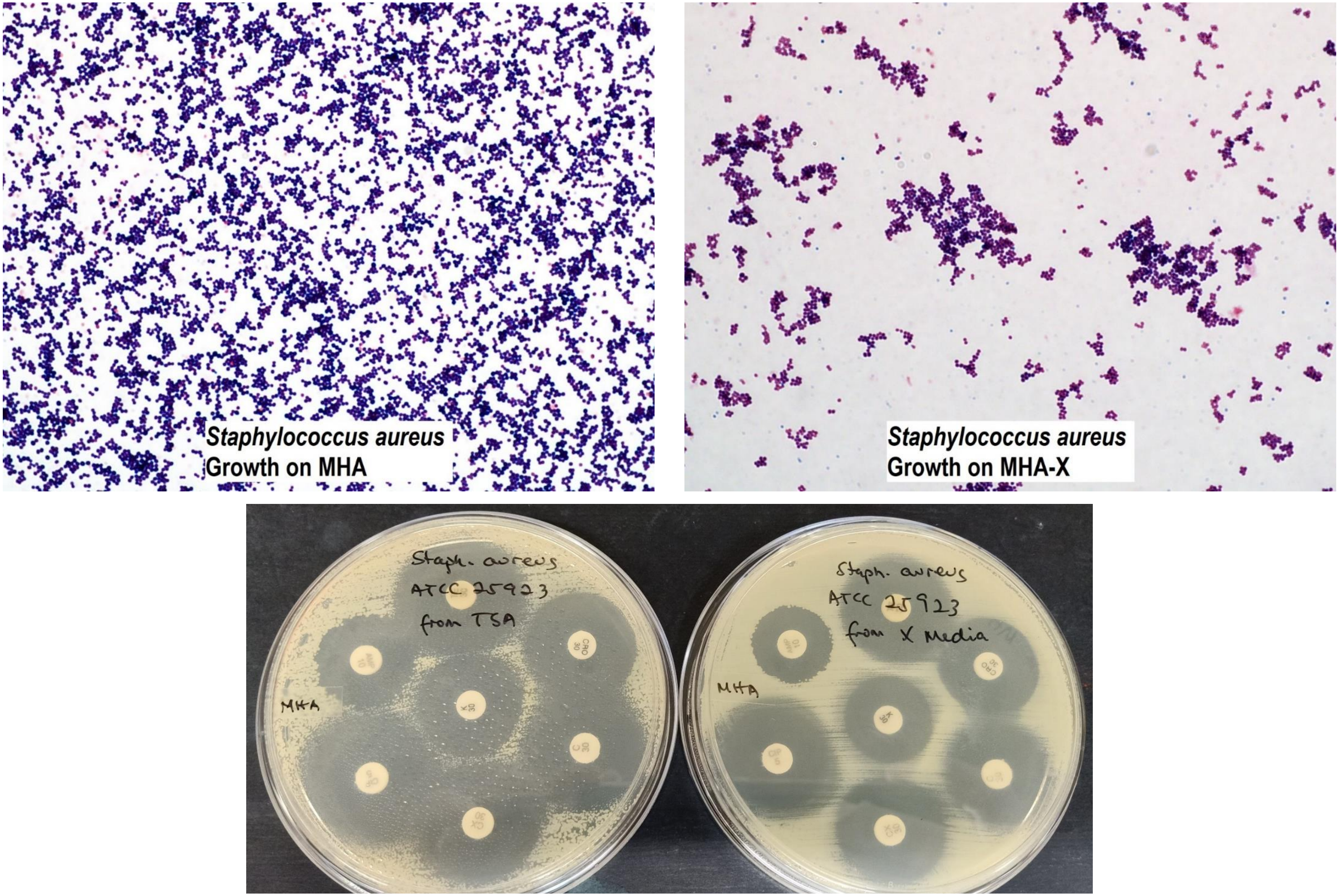

Figure 6. Gram staining of *Staphylococcus aureus* (SA) grown on (a) MHA, (b) MHA-C15; antibiotic sensitivity test of SA grown on (c) MHA and (d) MHA-C15.
